# Status and forecast of leprosy in the still endemic province of Formosa in northern Argentina

**DOI:** 10.1371/journal.pntd.0008881

**Published:** 2021-01-05

**Authors:** María R. Arnaiz, Mónica S. Iglesias, José I. Franco, Lucila Arzamendia, María S. Santini, Hugo C. Recalde

**Affiliations:** 1 Centro de Diagnóstico e Investigación en Endemo-epidemias, CeNDIE, Ciudad Autónoma de Buenos Aires, Argentina; 2 Departamento de Biodiversidad y Biología Experimental. Facultad de Ciencias Exactas y Naturales. Universidad de Buenos Aires, FCEyN UBA. Ciudad Universitaria, Ciudad Autónoma de Buenos Aires, Argentina; 3 Programa de Control de Leishmaniasis y Lepra de Formosa, PCLyL-F. Dirección de Medicina Preventiva y Prestaciones Especiales, Ministerio de Desarrollo Humano, Formosa, Argentina; Hospital Infantil de Mexico Federico Gomez, MEXICO

## Abstract

**Background:**

The province of Formosa, Argentina, is endemic for leprosy. In the present paper, we assessed the trend (T, 2002–2016 time series) and the forecast for 2022 of new case detection rate (NCDR) and determined the spatial distribution of new cases detected (NCD) of leprosy.

**Methodology/Principal findings:**

This is a descriptive observational study of 713 NCD of leprosy from provincial medical records between January 2002 and December 2016. The whole dataset from the provincial medical record was used to independently estimate the NCDR trends of the general population, age groups, sexes and Departments. This same database was used to estimate the NCDR forecast of the general population for 2022, applying a dynamic linear model with a local linear trend, using the MCMC algorithm. The NCDR was higher in men (*p*<0.05), increased with age (0.20, 8.17, 21.04, and 29.49 for the 0–14, 15–44, 45–64 and over 65-year-old age groups, respectively; *p*<0.05) and showed a downward trend (negative values) of estimated slopes for the whole province and each Department. Bermejo Department showed the highest (T:-1.02, 95%CI: [-1.42, -0.66]) and Patiño the lowest decreasing trend (T:-0.45, 95%CI: [-0.74, -0.11]). The NCDR trend for both sexes was similar (T:-0.55, 95%CI: [-0.64, -0.46]), and age groups showed a decreasing trend (S_15-44_:-103, S_45-64_:-81, S_>65_:-61, *p*<0.05), except for the 0–14 age group (S:-3, *p*>0.05), which showed no trend. Forecasts predicted that leprosy will not be eliminated by 2022 (3.64, 95%CI: [1.22, 10.25]).

**Conclusions/Significance:**

Our results highlight the status of leprosy in Formosa and provide information to the provincial public health authorities on high-risk populations, stressing the importance of timely detection of new cases for further elimination of the disease in the province.

## Author summary

Leprosy, a neglected tropical disease, is a public health problem in northern Argentina, causing permanent damage, stigmatization and discrimination. The Program for the Control of Leishmaniasis and Leprosy in Formosa province (PCLyLF) is responsible for the diagnosis and treatment of the disease and the active search for household contacts. The present study aimed to determine the spatial distribution of new cases of leprosy and the status of the disease using new case detection rate (NCDR) as the main epidemiological indicator. We determined the NCDR trends (2002–2016 time series) of the general population, age groups, sexes and Departments, and the NCDR forecast for the province by 2022. Our results indicated that leprosy was spread throughout Formosa, with higher rates in men and in patients over 65 years old. The NCDR showed a downward trend in the whole province and in each Department. The Departments of Bermejo and Matacos had the highest decreasing trend and the Department of Patiño the lowest one. Future projections show that leprosy will not be eliminated by 2022.

## Introduction

Leprosy is a chronic infectious disease caused by *Mycobacterium leprae*, affecting over 200,000 people each year worldwide and over 27,000 people in Latin America and the Caribbean [1]. Despite the low virulence of *M*. *leprae*, patients may suffer disabilities, stigmatization and mortality over time under unfavorable conditions such as low socio-economic status and concomitant infections [2]. In 2017 there was a decline in new cases with grade 2 disability (G2D) and the WHO set new targets for reducing the disease burden toward 2020 [3].

In Argentina, the earliest cases of leprosy were officially reported in 1792 and since then the number of patients increased gradually. The first map of leprosy distribution was published in 1928, considering the fluvial littoral coast, which includes the province of Formosa, as an endemic area [4]. Leprosy has been a compulsory notifiable disease since 1983 [5]. The elimination goal recommended by the WHO in 1991 was achieved in 2006 at a national level [6–7], except for Formosa and other northern provinces.

Formosa was considered to be National Territory in 1884 [8]. It was divided into the nine current Departments in 1915 [9] and reached the status of province in 1955 [10]. It has experienced a spectacular demographic growth from 19,281 to 113,790 inhabitants during the 1914–1947 inter-census period, with a high immigration flow from bordering countries endemic to leprosy. In the 1970s the demographic growth was favored by the improvement of two national routes that cross the province [11–12]. Despite the demographic growth trend, there was a high mortality rate due to neglected diseases such as leprosy, thus exposing the vulnerability of the public health system. Consequently, the Health Ministry of Formosa created the Leprosy Division in 1988 and then launched the Program for the Control of Leishmaniasis and Leprosy (PCLyL) [13]. The PCLyL of Formosa is responsible for the diagnosis, treatment and active surveillance of leprosy contacts. Although the epidemiological indicators have improved remarkably, the annual prevalence in the province is still higher than the national average (1.79/100,000 vs. 0.18/100, 000 inhabitants, respectively) and in addition, the PCLyL has diagnosed new cases in children and new cases of G2D in patients of different age groups [14].

Overall, the difficulty in reaching the goals for leprosy elimination in the province of Formosa are most likely related to the failure of early detection of new cases through active case-finding or management of household contacts. Nevertheless, the Global leprosy strategy 2016–2020 has been launched to accelerate actions toward a leprosy-free world [3]. It places special emphasis on strengthening patient and community awareness on leprosy by encouraging self-reporting. Likewise, household contacts of newly diagnosed leprosy patients should be encouraged to report voluntarily for examination [15].

In the province of Formosa, leprosy elimination is possibly hindered by different factors such as the climate [16], socio-cultural differences in the population (including Qom, Wichi and Guaraní peoples) [17], geographic barriers (i.e., extensive forests, swampy or flooded areas) and under-developed road infrastructures that limit the access of rural patients to health centers [18].

Based on the considerations mentioned above, knowledge of the leprosy status in Formosa is essential for the design of intervention strategies targeted to the high-risk population. The aims of the present study were to determine: 1) the status of leprosy in Formosa province; 2) the spatial distribution of new cases; 3) the NCDR trend for the 2002–2016 time series; and 4) the NCDR projections by 2022.

## Methods

### Ethic statement

The Research Ethics Committee of the CENAGEM-ANLIS, Dr. “Carlos G. Malbrán” Ministerio de Salud, Argentina, guaranteed by Dr. Cecilia Luna certified that patient data were stored safely and securely to ensure patient confidentiality and that the study raised no ethical concerns CAI-Arnaiz/2th June, 2017.

**Fig 1:**
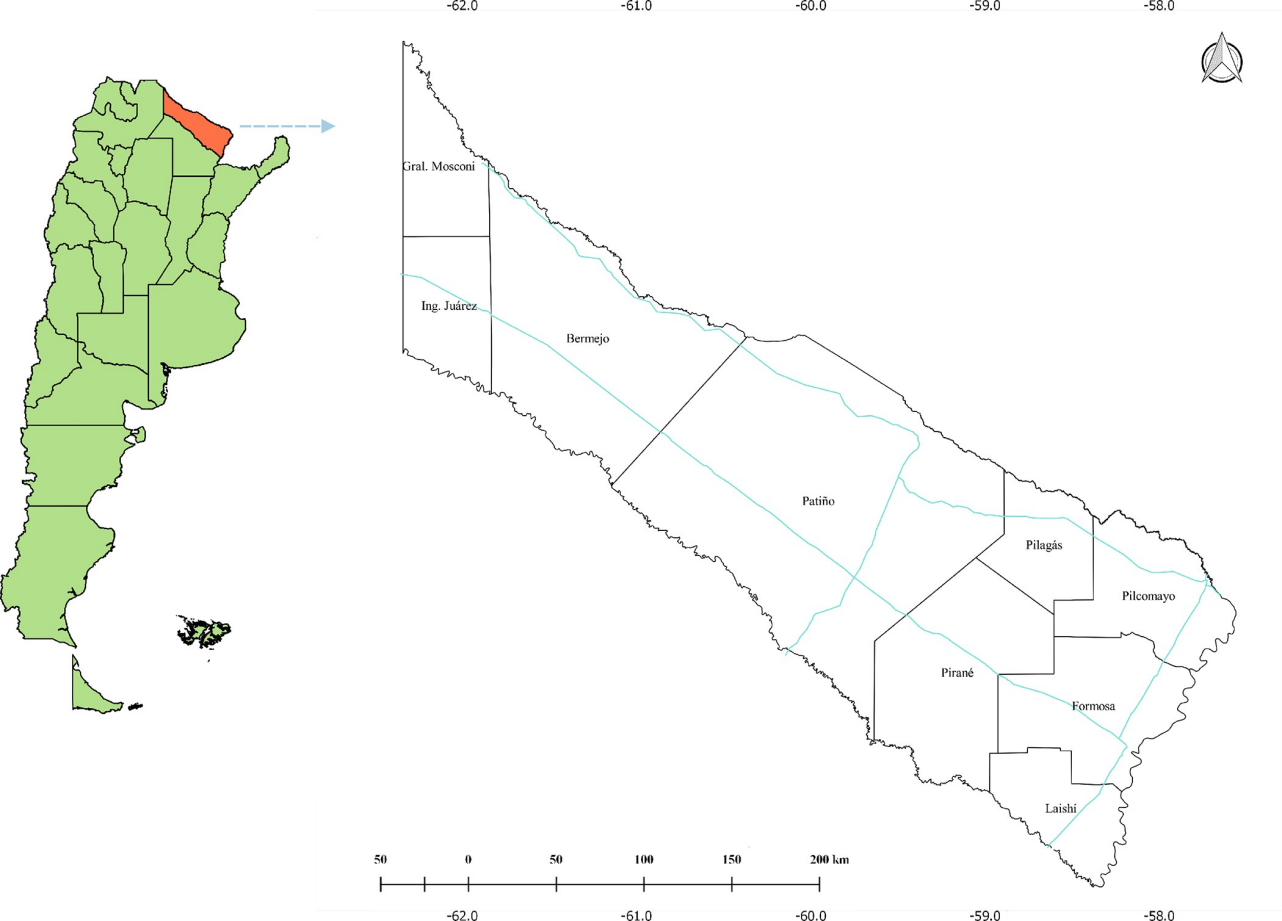
Map of Argentina indicating Formosa province. Map of Argentina with a magnification of the province of Formosa, which borders with Paraguay to the north and is connected with the rest of the country by National Routes 11, 86, and 81 (thin blue lines). Fig made with QGIS v.2.18.15 using data from the National Geographic Institute. Available at: http://www.ign.gob.ar/NuestrasActividades/InformacionGeoespacial/CapasSIG

Formal verbal consent was obtained for adult patients and for the parents of the child participants. Formosa (22–27°S, 57–63°W) covers 72,066 Km^2^ and is divided into nine Departments: Bermejo, Formosa, Laishi, Matacos, Patiño, Pilagás, Pilcomayo, Pirané, and Ramón Lista [9] (Fig 1).

In this descriptive observational study, data of 713 patients diagnosed with leprosy between 2002 and 2016 were obtained from the provincial medical records, which are centralized in the PCLyL. The variables retrieved were sex, age at the time of diagnosis by age group (0–14, 15–44, 45–64 and over 65 years old), year of diagnosis, and residential address and/or neighborhood.

To estimate the annual new case detection rates (NCDRs) we calculated the annual population size for the whole province, by age group and by sex and by Department for the time series 2002–2016. The population size of the intermediate and subsequent years to the censuses 2001 and 2010 were calculated assuming an exponential growth.

The NCDRs were re-calculated with the moving-average procedure centered on three points using the PAST statistical package version 3.2.0 [19].

New cases detected of leprosy (NCD) diagnosed in the province of Formosa between 2002 and 2016 were geo-referenced using the *Google Earth* open access tool (version 7.3.1), according to the address of residence/neighborhood. The geographic coordinates were plotted on a map constructed with SimpleMappr [20].

### Statistical analyses

The whole dataset from the provincial medical record was used to independently estimate the NCDR trends of the general population, age groups, sexes and Departments. This same database was used to estimate the NCDR forecast of the general population for 2022.

To assess the NCDR trend and forecast of Formosa general population, we fit a dynamic (structural) linear model [21–22] with local linear trend. The model was fit using an MCMC algorithm with 100,000 iterations, after discarding the first 10,000 iterations as burn-in. Data were transformed by f(x) = log(x + 1).

The local linear trend model (LLTM) is formulated as:ϵ

$$y_{t}=\mu_{t}+\epsilon_{t}$$

$$\mu_{t+1}=\mu_{t}+\delta_{t}+\eta_{0t}$$

$$\delta_{t+1}=\delta_{t}+\eta_{1t}$$

Where t is time expressed in years, **δ**_**t**_ is the slope of the local linear trend and **y**_**t**_ refers to the observed data. The error terms **ϵ**_**t**_ and **η**_**t**_ are Gaussian and independent of everything else. This model was used to predict the detection rate for 2022. Forecasting (2017–2022) was calculated as the median of the 90,000 repetitions and the confidence interval (CI) was based on 0.025 and 0.975 quantiles. The model was evaluated by the R-squared statistic, which is the proportion by which the residual variance is lower than the variance of the original observations and Harvey’s goodness-of-fit statistic. In this model, ν denotes the one-step ahead prediction error, n is the length of the series, and y is the original series.

The goodness-of-fit statistic is

$$1-\sum_{i=1}^{n}\nu_{i}^{2}/{\sum_{1=2}n\left(\Delta y_{i}-\Delta \bar{y}\right)}^{2}$$

and is analogous to R in a regression model, but the reduction in the sum of squared errors is relative to a random walk with a constant drift (y_t+1_ = y_t_ + β +ϵ_t_) which, according to Harvey (1989, equation 5.5.14) is a more relevant baseline than a simple mean. Unlike a traditional R-squared statistic, it can be negative. The LLTM presents a proportion of 68% by which the residual variance is less than the variance of the original observations. Harvey´s goodness-of-fit statistic was 0.28, thus indicating that the LLTM fit better than the baseline model. All analyses were performed with the bts [21] R package version 3.5.2.

To determine significant differences in NCDR among the four age groups (0–14, 15–44, 45–64 and over 65) were analyzed by means of the Friedman test followed by the Wilcoxon pairwise test. The ***A*** Mann-Kendall trend test for time series was performed for each age group. Analyses were made using PAST statistical package version 3.2.0 [19].

To assess differences in NCDR trends between sexes, we fit two linear mixed-effects regression models for longitudinal analysis (2002–2016) of NCDR at sex level, with time (in years) as a regressor. Our method corresponds to a Bayesian analysis with non-informative priors, because we lacked strong *a priori* information. We assumed a model with random effect for the intercept (model I) and a model with random effect for the slope and intercept (model II) for each sex. An unstructured correlation matrix was used in both models. The parameters of the models were estimated with the Markov Chain Monte Carlo (MCMC) method [23]. Four chains were run with 4,000 iterations each. The first 1000 iterations were discarded as burn-in, and the following 3,000 iterations were used to calculate the *a posteriori* distribution of parameters. The degree of convergence of a random Markov Chain was estimated using the Gelman-Rubin convergence statistic [24] (R-hat), based on the stability of outcomes between and within the chains; values close to 1 indicate convergence to the underlying distribution. The best model was chosen under the leave-one-out criterion (LOOIC). According to the LOOIC values obtained from models I and II, the model that best fit sex data was model I (with only random intercept). The R-hat statistic indicated good convergence (R-hat = 1).

To determine NCDR trends at Department level, we fit two linear mixed-effects regression models for longitudinal analysis (2002–2016) with time (in years) as a regressor. We used the same methodology as that used for estimating NCDR trends by sex (see above). The LOOIC values obtained from models I and II indicated that the model that best fit the Department data was model II (with random intercept and slope). The R-hat statistic indicated good convergence (R-hat = 1). The Department of Ramón Lista was excluded from the analysis because only one new case of leprosy was recorded during the study period.

Analyses by sex and Department were performed with the rstan and rstanarm packages in R software, version 3.5.*2*.

To evaluate significant differences in NCDRs between sexes for each Department (excluding Ramon Lista), we used the Wilcoxon test with 99,999 Monte Carlo simulations.

## Results

**Fig 2:**
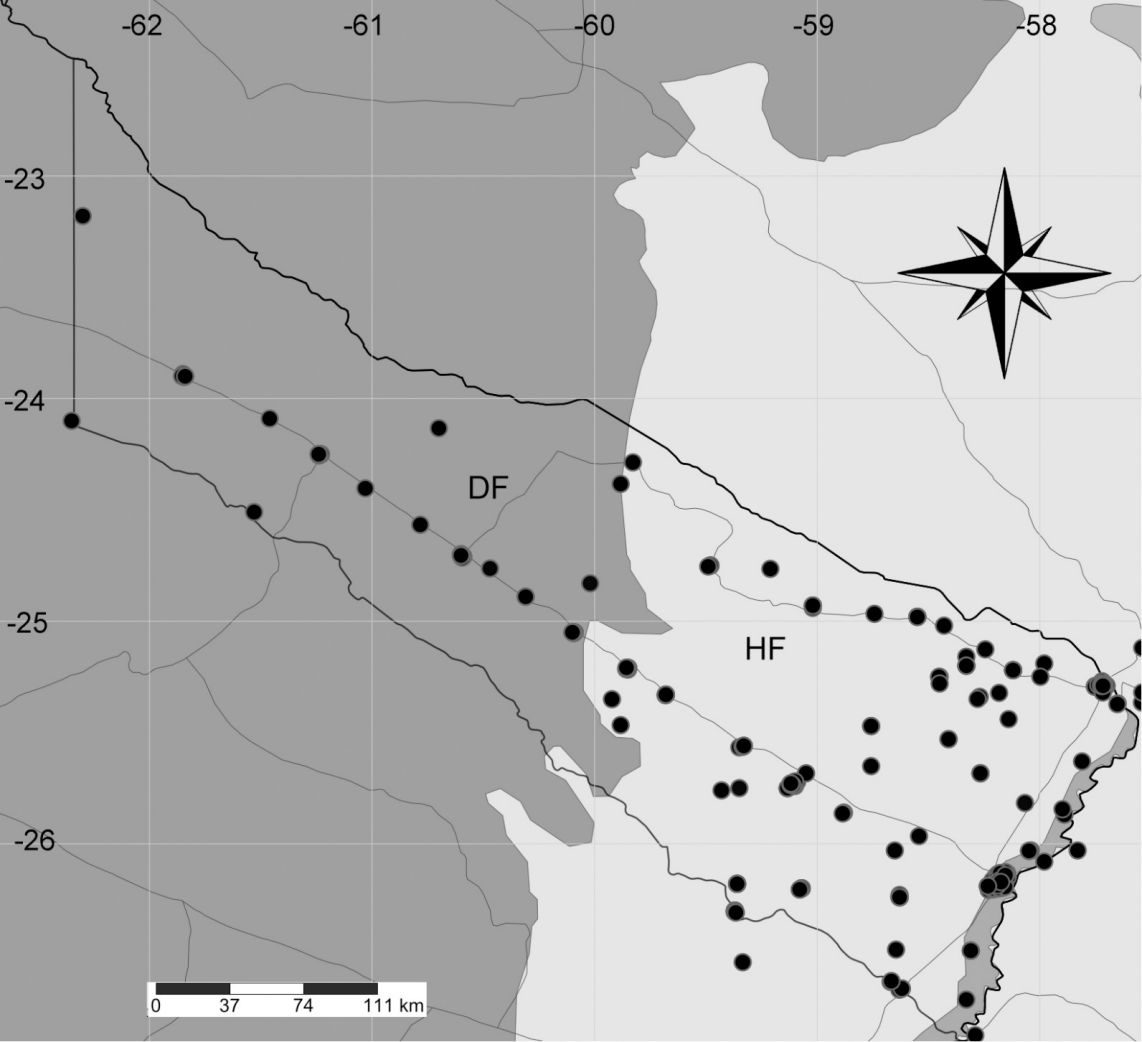
Geographic distribution of New Cases Detected (NCD) of leprosy in Formosa province. The map provides preliminary information and must be regarded just as an exploratory tool. Each point represents one or more NCD. Note that most of the points are distributed along National Routes 81 (in southeast-northwest direction) and 86 (running parallel to the Pilcomayo River and along the boundary with Paraguay). Map constructed with SimpleMappr [20].

According to the PCLyLF, a total of 713 NCD of leprosy were diagnosed between 2002 and 2016. These NCD were distributed throughout the province and located in eight municipalities along National Routes 81 and 86 (Fig 2). The number of NCD decreased between 2002 (n = 71) and 2016 (n = 32), in accordance with a significant decrease in NCDR from 13.3 in 2002 to 6.11

**Fig 3:**
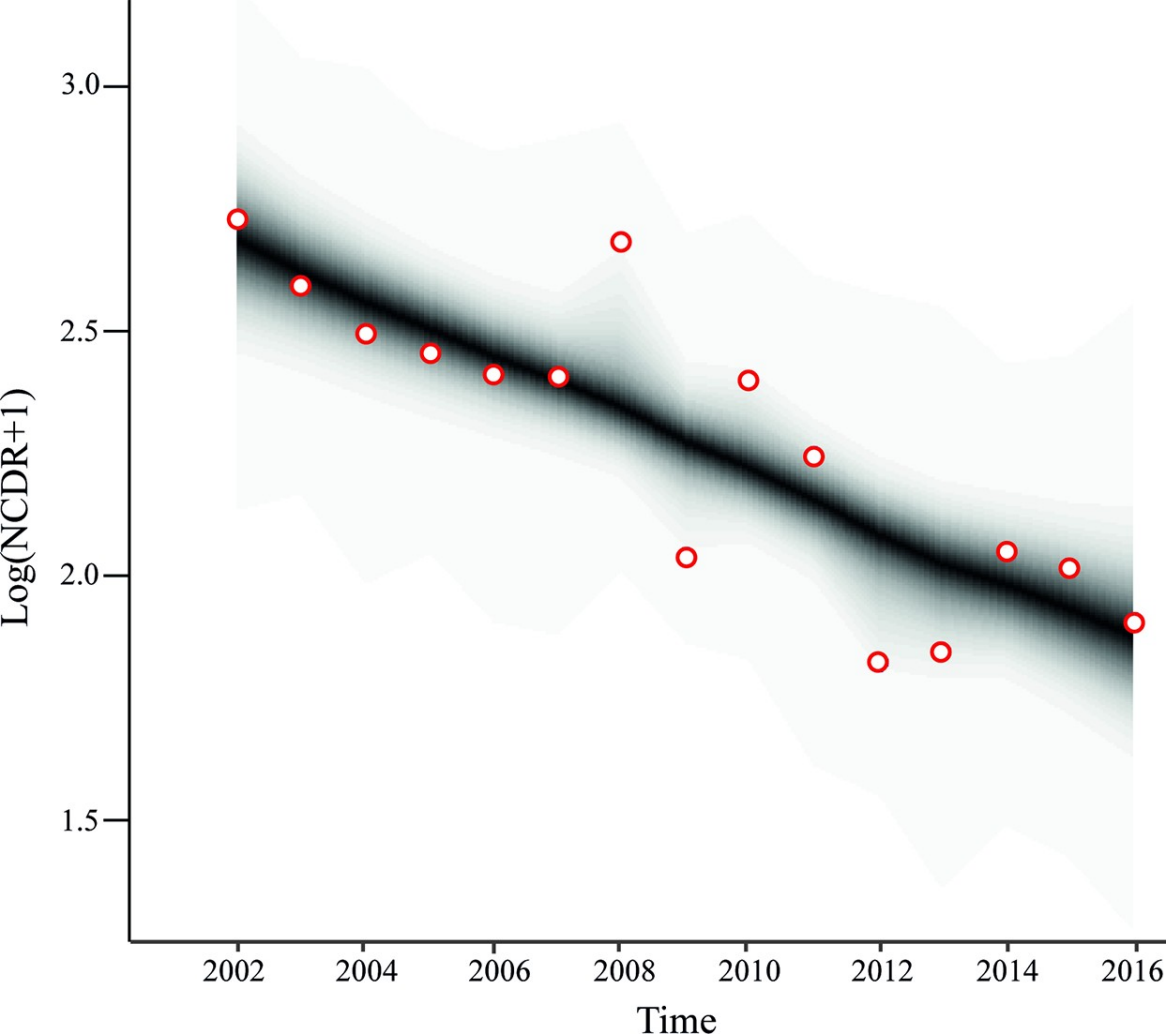
Trend of New Case Detection Rate (NCDR) as a function of time, for the time series 2002–2016. The graph shows the posterior distribution of NCDR as a function of time. Lighter to darker shades of blue represent the density levels of the parameter estimators from lower to higher, respectively. Circles indicate the observed NCDRs.

Fig 3 shows the posterior distribution of NCDRs after the implementation of the model as function of time for the general population. NCDR exhibited a downward trend between 2002 and 2016.

**Fig 4:**
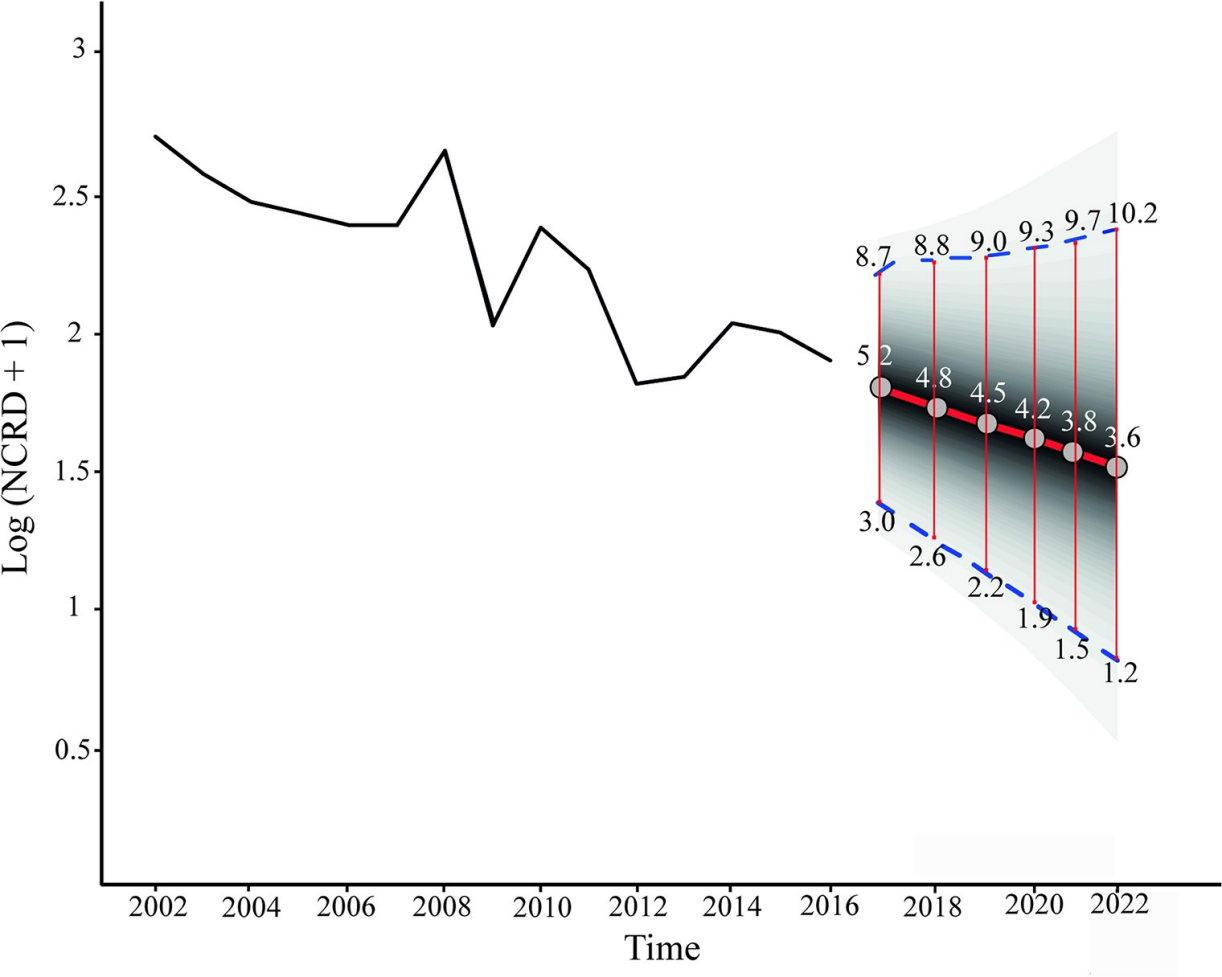
Forecast of New Case Detection Rate (NCDR) of leprosy in the province of Formosa between 2017 and 2022. Fig shows the original time series of NCDR as a function of time (solid black line). The forecast of NCDRs for 2017–2022 (to the right), presents the medians (circles) with their corresponding 95%CI (thin red lines) and the NCDR projected trend (thick red line). The future trend was constructed using the dynamic linear model with local linear trend. The confidence interval does not include zero suggesting that leprosy will not be eliminated by 2022.

The forecast with a horizon of six years predicted that NCDR will decrease in the province by 2022. However, taking into account that the zero is not included in the confidence interval despite its amplitude (3.64, 95%CI: [1.22–10.25]), we could predict with an accuracy of 95% that leprosy will not be eliminated by 2022 (Fig 4).

The analysis by age group showed significant differences in NCDR among the four groups (0–14: 0.20, 15–44: 8.17, 45–64: 21.04, over 65: 29.49, *p*<0.05 in all cases) (S1 Table), with NCDR increasing with age. All groups exhibited a decreasing trend between 2002 and 2016 (S_15-44_:-103, S_45-64_:-81, S_>65_ = -61, *p*<0.05 in all cases) except for the 0–14 age group (S = -3, *p*>0.05), which showed no trend.

**Fig 5:**
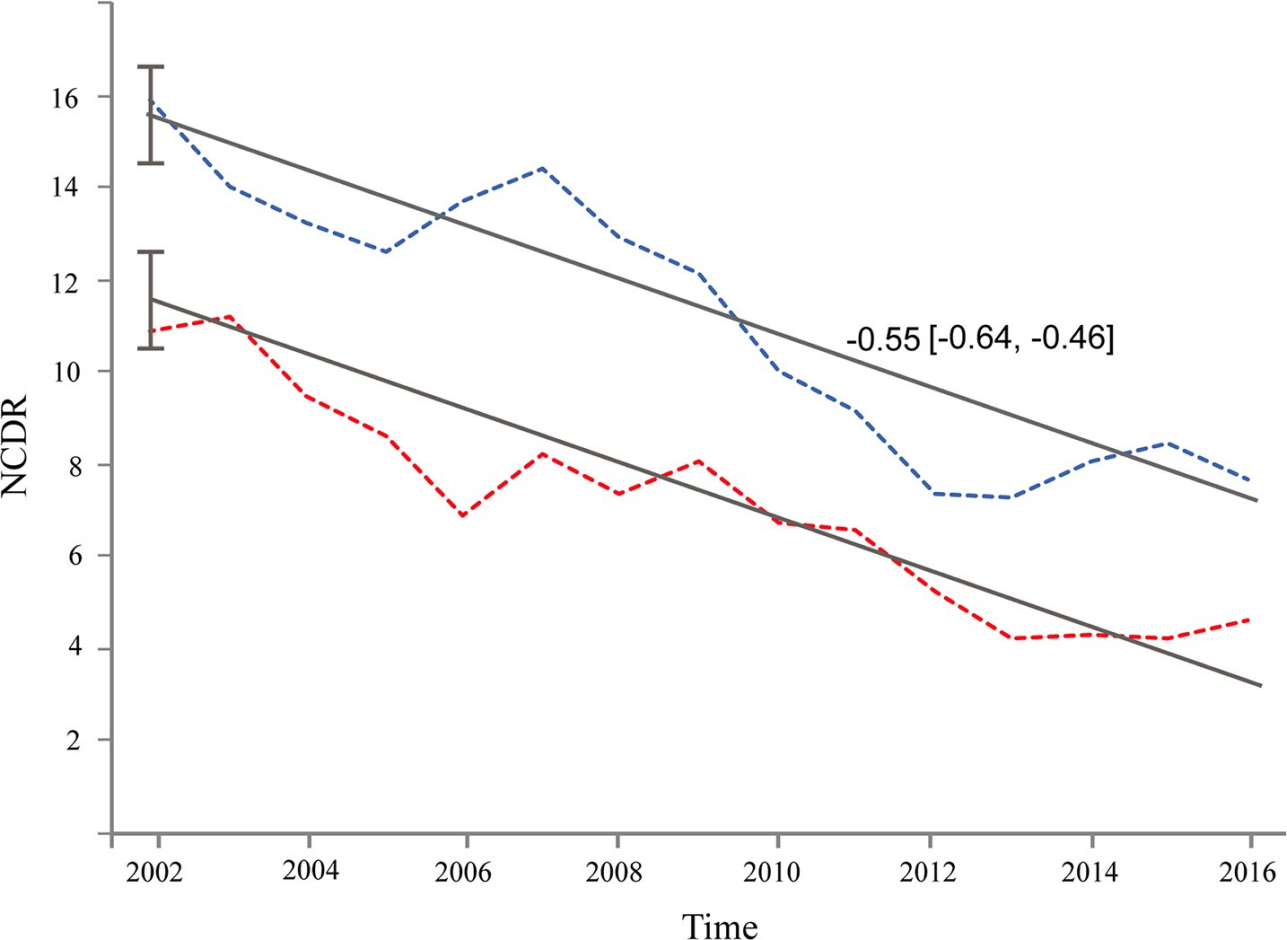
Trend of New Case Detection Rate (NCDR) in men and women as a function of time, for the time series 2002–2016. Fig 5 depicts the slopes and 95%CI of NCDR for men and women as a function of time. Although NCDR was higher in men (blue line) than in women (red line), its downward trend showed a similar speed for both sexes. The Model I was chosen (random intercept) under the leave-one-out criterion (LOOIC).

The analysis of the NCDR by sex for the whole province showed a higher rate for men than for women (12.17 and 6.86, respectively; *p*<0.05) (S1 Table). When analyzed by Department, the NCDR was higher for men in the Departments of Formosa and Matacos, while the opposite was found in the Department of Patiño, (6.82 and 11.94 for women and men, respectively, *p*<0.05) (Table 1). The downward trends in NCDR of men and women showed a similar speed, but the initial state (intercept) was higher in men (15.52, 95%CI: [14.59, 16.44]) than in women (11.55, 95%CI: [10.62,-12.46]) (Fig 5).

**Table 1:** New Case Detection Rate (NCDR) by sex and Department in Formosa province for the time series 2002–2016.

Department	Formosa	Pilcomayo	Laishi	Pilagás	Pirané	Patiño	Bermejo	Matacos
GENERAL	8.23 6.7–9.7	7.84 5.5–8.7	11.95 8.0–16.1	5.41 3.6–9.1	11.37 8.5–15.6	10.04 8.2–12.2	10.21 2.2–15.3	7.44 6.4–12.7
MEN	9.04* 7.3–11.1	6.5 3.2-9-1	15.22 7.5–19.1	6.89 3.5–10.7	7.20 5.1–19.3	6.82 5.7–10.6	9.52 2.1–14.3	9.77* 8.7–18.9
WOMEN	7.52 5.3–8.9	7.31 5.7–10.0	11.95 8.2–12.7	3.91 3.6–7.4	11.64 10.2–14.6	11.94* 10.1–15.4	5.17 0.0–11.0	4.88 0.0–7.9

The values represent the median (N = 15 in all cases) and the 25–75 percentiles of the NCDR for the population in each Department and discriminated by sex for the time series 2002–2016. Department of Ramón Lista was excluded from the analysis because only one new case of leprosy was recorded during the study period. Asterisk indicates significant differences in NCDR between sexes (Wilcoxon test with 99,999 Monte Carlo simulations, *p*≤0, 05).

**Fig 6:**
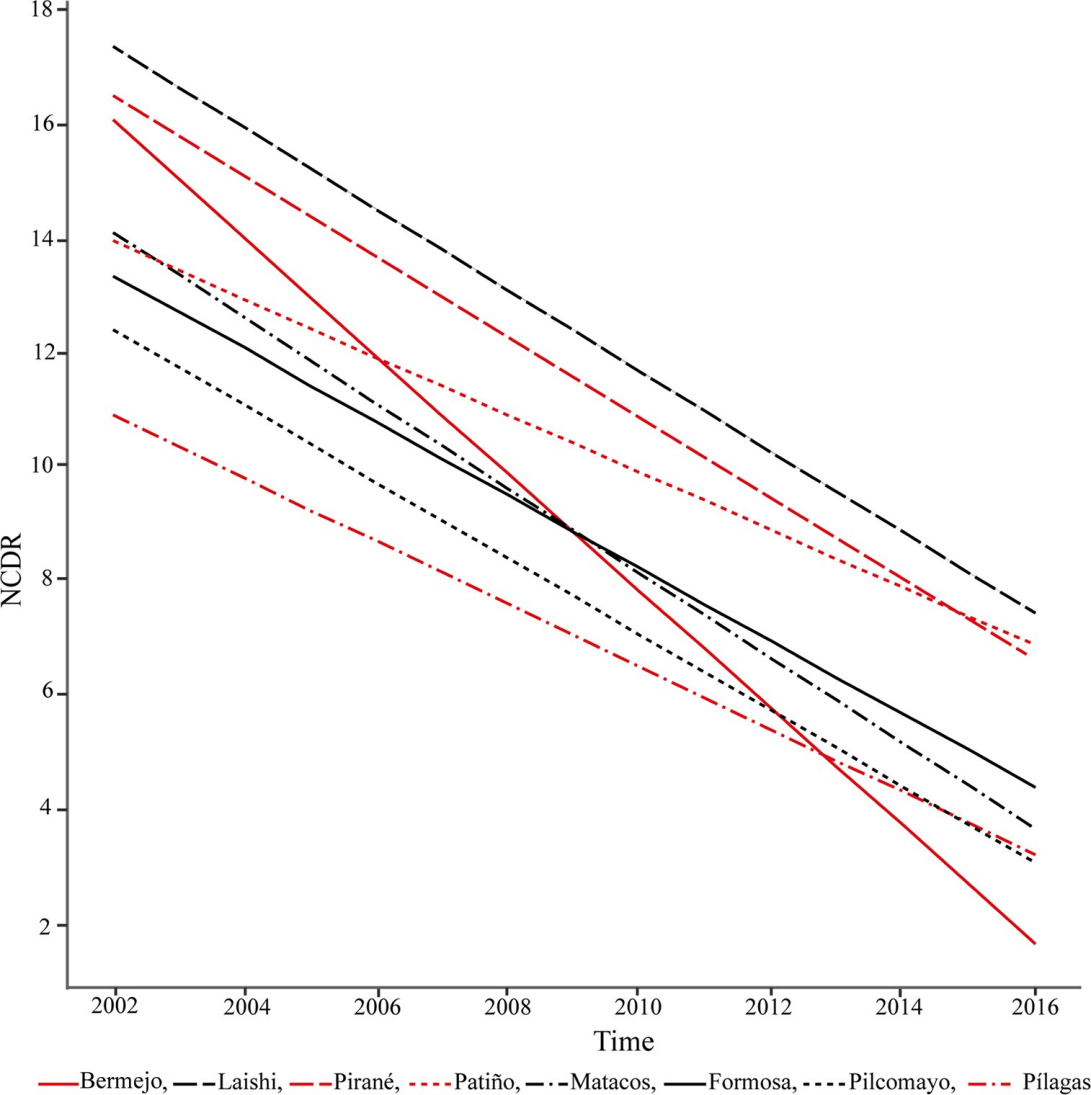
Trends of New Case Detection Rate (NCDR) in the Departments of Formosa as a function of time, for the time series 2002–2016. Fig 6 shows the trends of detection rates for the time series (2002–2016) in the Departments of Formosa province, except for the Department of Ramón Lista, where only one new case was detected. The Model II was chosen (random intercept and slope) under the leave-one-out criterion (LOOIC).

The estimated slopes of NCDR showed a decreasing trend for all the Departments, but at different rates Fig 6 and Table 2. Bermejo Department showed the highest (T:-1.02, 95%CI: [-1.42, -0.66]) and Patiño the lowest decreasing trend (T:-0.45, 95%CI: [-0.74, -0.11]).

**Table 2:** Trends of New Case Detection Rate (NCDR) for the Departments of Formosa.

Department	Trend	95% CI	R-hat
**BERMEJO** **(n = 18)**	-1.02	[-1.42, -0.66]	1
**FORMOSA** **(n = 298)**	-0.59	[-0.87, -0.27]	1
**LAISHI** **(n = 32)**	-0.69	[-1.02, -0.35]	1
**MATACOS** **(n = 18)**	-0.71	[-1.01, -0.43]	1
**PATIÑO** **(n = 106)**	-0.45	-0.74, -0.11]	1
**PILAGAS** **(n = 18)**	-0.49	[-0.84, -0.13]	1
**PILCOMAYO** **(n = 95)**	-0.61	[-0.92, -0.31]	1
**PIRANÉ** **(n = 116)**	-0.67	[-0.99, -0.36]	1

Median of the estimated slopes (Trends) of the NCDR by Department for the time series 2002–2016 with their confidence intervals (CI) based on 2.5% and 97.5% percentiles. R-hat values close to one indicate convergence to the underlying distribution. Twelve patients were excluded from the analysis because they could not be assigned to any Department.

## Discussion

This is the first systematic study on the epidemiology of leprosy in the province of Formosa in northern Argentina. We determined the current status of the disease, the distribution of new cases detected (NCD), and the trend and forecast for 2022 based on NCDR as epidemiological indicator. Formosa is historically endemic for leprosy and a recent national meeting of experts reported a prevalence of 1.64/100000 for the province [14]. In this context, the present work contributes with updated and useful information. Our results revealed that the disease is spread throughout the province and that there was a decreasing trend of NCDR between 2002 and 2016. Moreover, the forecast projected that NCDR will continue to decline by 2022, without disease elimination.

The mapping of new leprosy cases revealed that many of them were in towns (with health care centers) located along the national routes connecting Formosa with the rest of the country and Paraguay. In Formosa, only 27.3% of the road network is paved. The rest of the road segments are precarious and often flooded by rain leading to the isolation of a large part of the territory [18]. This factor may play an important role in late diagnosis and the under-reporting of new cases of leprosy [25], underlining the necessity for more flexible services of diagnosis and treatment, such as mobile medical offices [26].

In this paper, NCDR was higher in men than in women. Studies addressing sex and leprosy in different countries have also documented a lower prevalence/incidence of leprosy in women, pointing to stigmatization, less access to the health system, illiteracy and poor knowledge of the disease as major causes explaining under-reporting [27–28]. However, these reasons may not apply to Formosa, as women not only attend the health care center earlier than men but also take them to the doctor and monitor their compliance to the treatment (Dr. Hugo.C. Recalde, Director of the PCLyLF of Formosa, pers. comm.). On this basis, the lower NCDR in women may appear as a consistent finding. On the other hand, women may develop stronger immunological responses to *M*. *leprae* than men, as suggested by lower incidence and less severe clinical forms of disease in most areas of the world [29].

Our results indicated that the risk of leprosy increases with age, mainly affecting the elderly population over 65 years old. This may be related to the long current life expectancy (73.9 years old), increasing the chance of coming into contact with active transmission foci [30–31] and to the fact that symptoms may take several years to show up [32]. In regard to the latter, age-related problems such as lowering of the immune system, concomitant diseases and pathologies associated with the aging process may favor the onset of leprosy symptoms.

On the other hand, new G2D cases are an indicator of delayed detection of leprosy [33]. Unfortunately, we could not analyze this indicator because leprosy disability degrees were absent in many of the medical records from PCLyLF.

The sustained downward trend of leprosy from 2002 to 2016 in the whole province is most likely due to the systematized activities carried out by the PCLyLF, which possibly decreased the transmission of *M*. *leprae*. Although we found differences in the speed of NCDR trends among departments, all of them showed a decline over time. Such differences may be due to distinct performances of health care workers among Departments in terms of multidrug therapy implementation, surveillance and support to innovative actions [26]. Notwithstanding all this effort, our forecasting predicts that new cases of leprosy will continue to occur in the immediate future.

Our study suffers from the limitations imposed by the use of a secondary database not designed by us. It would have been useful to have additional information (e.g., socio-economic and environmental factors) to include in our analyses. Another constraint is represented by case under-reporting due to the long asymptomatic and/or oligosymptomatic stage of the disease, thus requiring trained professionals for diagnosis [34]. Moreover, case under-reporting may result from the difficult access to health care facilities in rural areas. On the other hand, the proportion of G2D is considered a strong indicator of late detection [33], but as mentioned above, the disability degree was sometimes absent from the medical record used in this study.

This epidemiological work represents a starting point to explore different aspects involved in the maintenance and transmission of leprosy in Formosa. It is a multicultural province including different indigenous peoples, with their own languages and ancestral knowledge. Our mapping of new cases detected reveals that leprosy is spread throughout the province, probably affecting different communities. In regard to the difference in incidence observed between men and women, it could be related to the role played by each gender in each culture. Undoubtedly, the understanding of the diverse social and cultural representations will help elucidate some underlying factors that hinder leprosy elimination.

In conclusion, our results indicate that leprosy persists as a serious public health problem in Formosa. The large number of new cases among the adult population and the projection showing that it will not be eliminated by 2022 are evidence of late diagnosis and the occurrence of active foci of transmission. Therefore, it is of great importance to develop specific control strategies targeted to high-risk populations and to intensify the active search for household contacts in areas with less access to public health centers. The information provided herein will allow health authorities to redirect efforts toward the elimination of leprosy.

## Supporting information

S1 STROBE Checklist STROBE checklist for this descriptive observational study. (DOCX)

S1 Table Number of new cases of leprosy (NCL) by sex and age group in time period 2002–2016 in Formosa. (XLSX)
